# Exploration of the Temporal Trends, Prevalence, and Factors of Acute Respiratory Infection or Diarrhoea: A Cross‐Sectional Analysis of Three National Surveys

**DOI:** 10.1002/hsr2.72529

**Published:** 2026-05-13

**Authors:** Mahdia Ahmed, Md Fuad Al Fidah, Mahabub Uz Zaman, Md Ridwan Islam, Tahmeed Ahmed, Mustafa Mahfuz

**Affiliations:** ^1^ Cornell University Ithaca New York USA; ^2^ Nutrition Research Division, icddr,b, Mohakhali Dhaka; ^3^ NIPSOM, Mohakhali Dhaka

## Abstract

**Background and Aims:**

Numerous interventions have been targeted to reduce the burden of acute respiratory infection (ARI) and diarrhoea in Bangladesh. Both remain the leading causes of mortality in children under 5 (U5C). We aimed to examine the temporal trends, prevalence, and factors associated with ARI and diarrhoea using three nationally representative surveys.

**Methods:**

We utilised data from three nationally representative surveys in Bangladesh: the Bangladesh Demographic and Health Survey (BDHS) 2017–2018, BDHS 2022, and the Multiple Indicator Cluster Survey (MICS) 2019. Analyses included the Cochran–Armitage test for trend, a multiple‐adjusted binomial logistic regression model, slope and relative indices of inequality (RII), and the Mantel–Haenszel test with an approximate test of homogeneity of odds ratios. A *p*‐value of < 0.05 was considered statistically significant.

**Results:**

A total of 39,919 U5C were included for ARI and 39,875 U5C for diarrhoea. A downward, monotonic trend was observed in ARI prevalence from 3.1% in 2017–2018 to 1.4% in 2022. In contrast, diarrhoea prevalence increased from 4.9% in 2017–2018 to 6.9% in 2019 and declined to 4.8% in 2022. Male children were associated with higher odds of ARI (aOR: 1.37, 95% CI: 1.04–1.82), and younger children were associated with lower odds of diarrhoea (aOR: 0.97, 95% CI: 0.96–0.98 in 2017–2018). Relative inequality remained significant for both ARI (RII: 2.36 in 2017–2018) and diarrhoea (RII: 1.92 in 2022), indicating disparities across wealth groups. Heterogeneity across survey years was observed only for the association of underweight status and wealth index with diarrhoea.

**Conclusion:**

ARI prevalence declined by a 2.2‐fold over 5 years, suggesting effectiveness of existing child health strategies, including immunisation and IMCI. Diarrhoea showed no consistent decline. Risk remained higher among male and younger children, with persistent socioeconomic inequalities. Steps should also be taken to consider wealth‐related accessibility and feasibility for future policy‐focused recommendations.

## Synopsis

Study question:

- What is the temporal trend, prevalence, and factors associated with ARI and diarrhoea across three nationally representative surveys?

What's already known?

- ARI and diarrhoea remain two of the top leading causes of under‐5 mortality in Bangladesh.
- WASH, education, and income have been identified as significant determining factors for ARI or diarrhoea in previous survey years; however, no trend has been determined.

What does this study adds?

- Provides an analysis of ARI and diarrhoea across three survey years, including an assessment of the determinants.
- Includes data from the latest national data set available.
- Applied SII and RII to visualise diarrhoea‐related socioeconomic inequalities in children under 5 using three of the latest datasets.

## 1 Introduction

Diarrhoea and acute respiratory infection (ARI) are leading global disease burdens among under 5 children (U5C) worldwide. Diarrhoea remains the third leading cause of U5C death. Globally, ARI causes 20% of mortality in U5C, with Southeast Asia having the highest incidence of ARI, followed by sub‐Saharan Africa [1, 2].

The United Nations (UN)'s Sustainable Development Goal 3.2.1 targets under‐5 mortality reduction to at least as low as 25 per 1000 live births [3]. Global U5C mortality decreased by 59% from 1990 to 2019 [4]. However, recent UNICEF data show that Bangladesh has an under‐5 mortality rate of 30.6 per 1000 live births, above the SDG target [5]. Therefore, although under‐5 mortality has steadily declined since 2011, it remains a major public health challenge in the country [6]. Two of the leading causes of under‐5 mortality in Bangladesh include pneumonia and diarrhoea [7]. A 2021 study found that diarrhoea affects one in 20 children, and ARI affects one in three [3]. Furthermore, ARI prevalence has risen from 39% to 46% from 2014 to 2019 [7]. Similarly, for diarrhoea, as of 2022, there was a 5.7% prevalence among U5C, which was reported by one study.

Existing research has used nationally representative data from Bangladesh to investigate the prevalence and determinants of diarrhoea and ARI in U5C. For example, in 2024, a study was conducted to analyse the temporal trends in diarrhoea for children under 5 using MICS 2006–2019, highlighting a spike in prevalence to 8.8% in 2019 [8]. Similarly, Rahman and Hossain conducted a study using BDHS 2017–2018 and found that 4.7% had diarrhoea and 35.8% had ARI, and evaluated determinants such as poor sanitation, low maternal education, and wealth index [9].

However, limited studies have evaluated a longitudinal analysis using all three of the latest datasets (Bangladesh Demographic and Health Survey (BDHS) 2017–2018, 2022, and Multiple Indicator Cluster Survey (MICS) 2019). Additionally, there are limited studies that analysed the same determinants for both diarrhoea and ARI concurrently in one study using the latest available nationally representative data, with an additional focused analysis on wealth inequality using both slope index inequality (SII) and relative index inequality (RII). Therefore, to address these gaps, this study aimed to examine the prevalence and determinants of ARI and diarrhoea using three nationally representative surveys (BDHS 2017–2018, MICS 2019, and BDHS 2022) with an analysis of the wealth index inequality.

## 2 Methods

### 2.1 Data Overview

**FIGURE 1:**
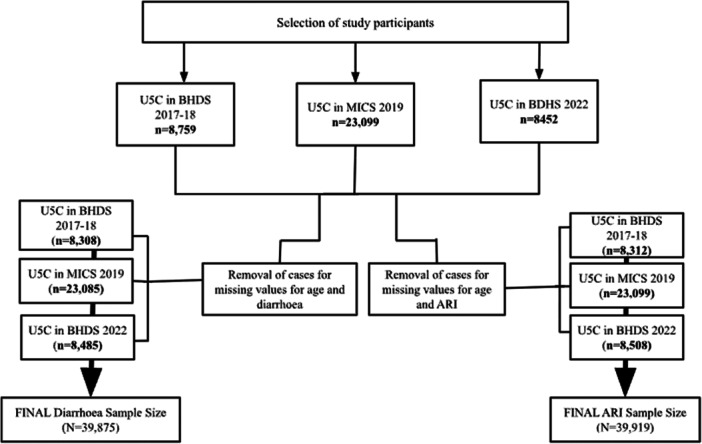
Schema representing the sample selection of children under 5 with either diarrhoea or acute respiratory infection (ARI). BDHS, Bangladesh Demographic and Health Survey; MICS, Multiple Indicator Cluster Survey.

This study utilises nationally representative data from three surveys: the Bangladesh Demographic and Health Survey (BDHS) 2017–2018, the Multiple Indicator Cluster Survey (MICS) 2019, and BDHS 2022. BDHS is a nationally representative household survey that has been conducted approximately every 3–5 years since the 1990s, while MICS is a household survey programme supported by UNICEF and implemented periodically [10, 11, 12]. The survey years selected for this study reflect the most recent publicly available and comparable datasets at the time of data collection. Data from 2021 to 2022 were not available for MICS and the MICS surveys are also conducted periodically. All three surveys include demographic information and health and nutrition indicators for U5C. All three surveys employed a two‐stage stratified sampling design to assess key demographic and health indicators. From a predetermined number of census enumeration areas that were systematically selected using the probability proportional to size method, 20 households were selected from each. Previous studies have also combined data from MICS and BDHS for analysis [13, 14, 15]. The 2017–2018 BDHS included 8759 U5C in the survey, the MICS 2019 included 23,099 U5C, and the 2022 BDHS included 8784 U5C. Additional details of the survey methodology, sample design, and data collection tools are available elsewhere [10, 11, 12]. U5C that were surveyed from the three datasets were included in this study. Cases with missing information on age or disease status for ARI or diarrhoea were excluded. Figure 1 further details the sample collection methodology. A total of 39,919 children under 5 were included for ARI and 39,875 for diarrhoea. The prevalence of ARI was 3.1% in 2017–2018, 2.0% in 2019, and 1.4% in 2022, while diarrhoea prevalence was 4.9%, 6.9%, and 4.8%, respectively. The study population was evenly distributed by sex, with approximately half male children, and the median age ranged from 28 to 30 months across survey years.

### 2.2 Operational Definition of the Outcome Variables

In this study, the following two outcome variables were assessed: ARI and diarrhoea.

### 2.3 ARI

We defined ARI based on the mother's report that the child had a cough and was either breathing faster than normal with short, quick breaths or having difficulty breathing due to a chest problem. Children who had only a blocked nose in the past 2 weeks were not considered to have ARI. The U5C were classified as ‘Present’ if they had symptoms of ARI, and ‘Absent’ if they did not within 2 weeks before the interview [16].

### 2.4 Diarrhoea

Diarrhoea is defined as the passage of 3 or more loose or liquid stools per day, or more frequently than is normal for the individual [9]. Presence of Diarrhoea was categorised as ‘yes’ or ‘no’ using the answer from a question that asked whether a child aged 5 years or less had experienced Diarrhoea within 2 weeks before the interview [2].

### 2.5 Independent Variables

Variables were selected and recorded based on previous studies and available information in three nationally representative datasets [3, 17, 18].

### 2.6 Child‐Level Independent Variables

The following variables were considered: age in months, sex (male/female), residence (urban/rural), stunting (absent/present), wasting (absent/present), and underweight (absent/present).

### 2.7 Parental‐Level Independent Variables

The parental‐level independent variables were mother's education level (none or up to primary/primary/secondary/post‐secondary), mother's media exposure (yes/no), age of the mother (in completed years), age of the father (in completed years), age of household head (in completed years), and sex of household head (male or female).

### 2.8 Household‐Level Independent Variables

We considered the following variables as the independent variables related to the number of U5C in the household, wealth index (poorest, poorer, middle, richer, and richest), toilet facilities shared with other households (no/yes), and type of toilet (unimproved/improved).

### 2.9 Stunting, Wasting, and Underweight

Stunting, wasting, and underweight were defined using the WHO criteria. Stunting was considered present if the height/length‐for‐age z score was <−2 standard deviations (SDs), wasting (present) if the weight‐for‐height/length z score was <−2 SDs, and underweight (present) if the weight‐for‐age z score was <−2 SDs [19].

### 2.10 Mother's Media Exposure

The variable ‘maternal media exposure’ was constructed by summing three indicators: the frequency of reading newspapers or magazines, listening to the radio, and watching television. Following prior literature, this variable was categorised into two groups: ‘not exposed’ (total score = 0) and ‘exposed’ (total score > 0) [20].

### 2.11 Type of Toilet

Improved toilet sources, according to WHO, include flush or pour‐flush to a piped sewer system, septic tank pit latrines, ventilated‐improved pit latrines, or pit latrines with slab or composting toilets. Unimproved toilet services, according to WHO, are defined as shared or public‐use sanitation facilities, flush or pour‐flush toilets elsewhere [17].

### 2.12 Wealth Index

The wealth index is a composite indicator of household socioeconomic status derived using principal component analysis (PCA) on asset ownership and housing characteristics. Variables typically include durable goods, housing materials, water sources, sanitation, and access to utilities. Each variable is standardised, and PCA is applied to generate weights, with the first principal component retained as it explains the largest variation [21]. Household scores are calculated as weighted sums of these variables and then used to rank households. The population is subsequently divided into quintiles, from poorest to richest, following previous studies [18].

### 2.13 Statistical Analysis

All statistical analyses were conducted using R (version 4.5.1; https://www.R-project.org/) and independently replicated in STATA 19 (StataCorp, College Station, TX, USA) to ensure analytical robustness. Descriptive statistics were applied to summarise the data. Categorical variables were expressed as frequencies and percentages, while continuous variables were reported as medians with interquartile ranges (IQRs) after confirming their non‐normal distribution using the Shapiro–Francia test. The prevalence of ARI and diarrhoea was estimated separately for each survey round. Temporal trends in the prevalence of ARI and diarrhoea across the survey years were assessed using the Cochrane–Armitage test for trend. The Cochran–Armitage test for trend assesses whether there is a linear change in proportions across ordered groups, such as survey years. It is used for binary outcomes and tests for a monotonic increase or decrease in prevalence. The null hypothesis assumes no trend, while the alternative assumes a linear trend across categories [22, 23]. A multiple binomial logistic regression model was developed to estimate the adjusted odds ratios (AORs) with 95% confidence intervals (CIs) for the associations between each independent variable and the outcomes. Collinearity was checked using the variable inflation factor (VIF). A VIF of > 5 was considered significant. Adjusted models were built using all independent variables.

Mantel–Haenszel stratified analysis was used to estimate the stratum‐adjusted pooled odds ratios for unadjusted associations. The approximate test of homogeneity of odds ratios was applied to evaluate the homogeneity of odds ratios across the three survey periods. Additionally, to examine socio‐economic inequalities in the distribution of ARI and diarrhoea, both absolute and relative inequality measures were computed using the Slope Index of Inequality (SII) and the Relative Index of Inequality (RII). The SII represents the absolute difference in predicted prevalence between the most disadvantaged and most advantaged groups, whereas the RII represents the relative ratio of prevalence between these groups. Further details regarding the SII and RII can be found elsewhere [22].

Sampling weights were not applied, as the analysis involved pooling data from multiple surveys with differing sampling designs and weighting structures. Applying a unified weighting approach across BDHS and MICS was not feasible; therefore, analyses were conducted using unweighted pooled data. All tests were two‐sided, and a *p*‐value of < 0.05 was considered statistically significant.

## 3 Results

**FIGURE 2:**
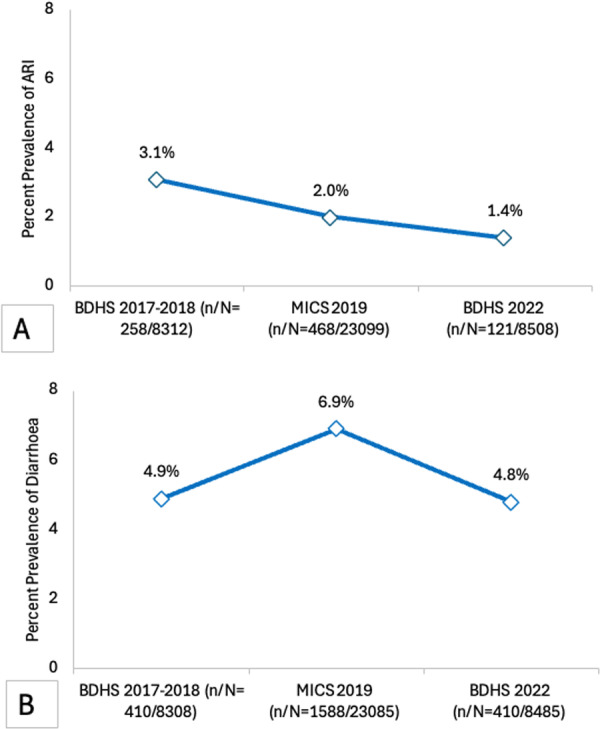
Temporal trends in the prevalence (%) of (A) prevalence of ARI: *p*‐value for Cochrane–Armitage test < 0.001, *p*‐value for test of departure from trend = 0.11. (B) Prevalence of diarrhoea among children under 5 across BDHS 2017–2018, MICS 2019, and BDHS 2022. Data points represent survey‐specific prevalence estimates. For ARI, the trend test was significant (*p* < 0.001) with no evidence of departure from linearity (*p* = 0.11). For diarrhoea, no significant monotonic trend was observed (*p* = 0.73), and the test of departure from linear trend was also non‐significant (*p* = 0.11).

The prevalence of ARI showed a downward trend over the survey years. In 2017–2018, the prevalence of ARI was 3.1%, which declined to 2.0% in 2019, and then further declined in 2022 to a prevalence of 1.4%. The Cochrane–Armitage test supported this to be a significant downward trend (*p* < 0.001). Furthermore, a test of departure from trend was found to not be significant, suggesting that the decline followed a linear pattern (*p* = 0.105). Figure 2B shows the trend of prevalence of diarrhoea across three survey years. In 2017–2018, the prevalence of diarrhoea was 4.9%, then increased in 2019 to 6.9%, but decreased to 4.8% by 2022 (Figure 2).

Across the three surveys, ARI prevalence declined from 3.1% in 2017–2018 to 2.0% in 2019 and 1.4% in 2022. Male children consistently had a higher prevalence than females in all years (*p* = 0.006 in 2017–2018; *p* < 0.001 in 2019; *p* = 0.01 in 2022). Age was also significantly associated with ARI across all surveys (*p* < 0.001 in 2017–2018; *p* < 0.001 in 2019; *p* = 0.01 in 2022). Wealth index was significantly associated in 2017–2018 where among the ARI cases, 29.5% were from the poorest vs. 14.7% from the richest (29.5% vs. 14.7% in the richest, *p* = 0.02), but not in later surveys. Maternal media exposure was protective in 2017–2018 (55.4% vs. 64.3%, *p* = 0.01) but not thereafter. Additional factors showed year‐specific associations, such as parental age (*p* = 0.01 for mothers, *p* = 0.01 for fathers) and shared toilet use (*p* = 0.03) in 2019, and maternal secondary education in 2022 (*p* = 0.05) (Table 1).

**TABLE 1:** Frequency and distribution of factors associated with ARI.

Variables	BDHS 2017–2018			MICS 2019			BDHS 2022		
	Total *n* (%)	ARI	*p* value	Total *n* (%)	ARI	*p* value	Total *n* (%)	ARI	*p* value
		Yes *n* (=248) (% = 3.1)*			Yes *n* (=468) (% = 2.0)*			Yes, *n* (=121) (% = 1.4)*	
Child's age in months	28 (14.0–44.0)	20 (10–41)	< 0.001	30 (15.0–44.0)	20 (9–36)	< 0.001	29.0 (13.0–44.0)	24 (12–35)	0.01
Mother's age in years	25 (21.0–30.0)	24.5 (20–29)	0.15	26 (23.0–31.0)	26 (22–30)	0.01	26.0 (22.0–30.0)	26 (22–32)	0.79
Father's age in years (*n* = 8176)	33 (29.0–38.0)	33 (28–38)	0.26	34 (30.0–39.0)	33 (29–38)	0.00	34.0 (29.0–39.0)	34 (30–40)	0.75
Household head's age in years	39 (30.0–54.0)	40 (31–52)	0.71	38 (32.0–52.0)	37 (31–50)	0.13	40.0 (32.0–54.0)	40 (32–55)	0.39
Number of U5C in the household	1.0 (1.0–2.0)	1.0 (1.0–2.0)	0.68	1.0 (1.0–2.0)	1.0 (1.0–1.0)	0.09	1.0 (1.0–2.0)	1.0 (1.0–2.0)	0.28
Sex (male), *n* (%)	4329 (52.1)	156 (60.5)	0.01	11,950 (51.7)	284 (60.7)	< 0.001	4361 (51.3)	77 (62.0)	0.01
Residence (urban), *n* (%)	2871 (34.5)	83 (32.2)	0.42	4303 (18.6)	87 (18.6)	0.98	2804 (33.0)	31 (25.6)	0.08
Maternal level of education			0.18			0.79			0.05
No education	592 (7.1)	13 (5.0)		2594 (11.2)	49 (10.5)		529 (6.2)	6 (5.0)	
Primary	2386 (28.7)	83 (32.2)		5563 (24.1)	121 (25.9)		2033 (23.9)	27 (22.3)	
Secondary	3916 (47.1)	127 (49.2)		11,356 (49.2)	229 (48.9)		4410 (51.8)	76 (62.8)	
Post‐secondary	1418 (17.1)	35 (13.6)		3586 (15.5)	69 (14.7)		1536 (18.1)	12 (9.9)	
Shared toilet (yes), *n* (=7283) (%)	2535 (34.8)	82 (36.9)	0.50	6393 (28.2)	150 (32.8)	0.03	2217 (29.1)	33 (32.4)	0.46
Sex of the household head (male), *n* (%)	7274 (87.5)	225 (87.2)	0.88	21,272 (92.1)	443 (94.7)	0.04	7477 (87.9)	106 (87.6)	0.93
Maternal media exposure (exposed), *n* (%)	5320 (64.0)	143 (55.4)	0.00	14,313 (62.6)	293 (63.3)	0.74	4825 (56.7)	74 (61.2)	0.32
Type of toilet (improved), *n* (=7354) (%)	4726 (64.3)	136 (59.7)	0.14	15,233 (66.0)	309 (65.8)	0.95	6118 (79.9)	79 (76.0)	0.32
Wealth index			0.02			0.70			0.53
Poorest	1826 (22.0)	76 (29.5)		5755 (24.9)	123 (26.3)		1802 (21.2)	24 (19.8)	
Poor	1667 (20.1)	56 (21.7)		4838 (20.9)	107 (22.9)		1712 (20.1)	29 (24.0)	
Middle	1482 (17.8)	42 (16.3)		4352 (18.8)	81 (17.3)		1695 (19.9)	27 (22.3)	
Rich	1637 (19.7)	46 (17.8)		4310 (18.7)	83 (17.7)		1649 (19.4)	24 (19.8)	
Richest	1700 (20.5)	38 (14.7)		3844 (16.6)	74 (15.8)		1650 (19.4)	17 (14.1)	
Stunting (present), *n* (=7849) (%)	2461 (31.4)	87 (34.9)	0.22	5965 (27.2)	143 (32.1)	0.02	966 (23.5)	13 (23.2)	0.96
Wasting (present), *n* (=7831) (%)	667 (8.5)	26 (21.3)	0.28	2175 (9.9)	46 (10.3)	0.77	483 (11.8)	7 (12.7)	0.82
Underweight (present), *n* (=8050) (%)	1806 (22.4)	55 (21.7)	0.76	5116 (22.8)	118 (26.1)	0.09	946 (22.8)	12 (21.4)	0.81

* Column % may not add up to 100% due to rounding. Percentages in the outcome column represent the proportion of children with ARI within each covariate category.

Across all surveys, younger age was consistently associated with lower odds of ARI (2017–2018: aOR = 0.98, 95% CI 0.98–0.99; 2019: aOR = 0.98, 95% CI 0.97–0.98; 2022: aOR = 0.98, 95% CI 0.96–1.00). Male sex was consistently associated with higher odds of ARI (2017–2018: aOR = 1.37, 95% CI 1.04–1.82; 2019: aOR = 1.47, 95% CI 1.20–1.79; 2022: aOR = 1.93, 95% CI 1.05–3.55). Maternal education was also associated with higher odds in 2017–2018 for primary education (aOR = 2.81, 95% CI 1.27–6.21) and secondary education (aOR = 2.99, 95% CI 1.34–6.65). This pattern was not evident in 2019 or 2022. Shared toilet use was associated with higher odds in 2019 (aOR = 1.27, 95% CI 1.02–1.57), but there was not a similar pattern in other survey years. Having more under‐5 children in the household was also associated with ARI in 2019 (aOR = 0.82, 95% CI 0.67–1.00) (Table 2).

**TABLE 2:** Logistic regression of factors associated with ARI.

Variable	Categories	BDHS 2017–2018 (*n* = 6740)		MICS 2019		BDHS 2022	
		aOR (95% CI)	*p* value	aOR (95% CI)	*p*‐value	aOR (95% CI)	*p* value
Child's age in months		0.98 (0.98–0.99)	< 0.001	0.98 (0.97–0.98)	< 0.001	0.98 (0.96–1.00)	0.01
Mother's age in years		1.00 (0.96–1.04)	0.98	1.00 (0.97–1.03)	0.95	0.99 (0.93–1.06)	0.85
Father's age in years		1.01 (0.98–1.04)	0.58	0.99 (0.97–1.01)	0.40	1.05 (1.00–1.10)	0.07
Household head's age in years		1.01 (1.00–1.02)	0.26	1.00 (0.99–1.01)	0.76	0.99 (0.97–1.02)	0.58
Number of U5C in the household		0.92 (0.74–1.15)	0.46	0.82 (0.67–1.00)	0.05	1.30 (0.88–1.93)	0.19
Sex	Female	Ref.		Ref.		Ref.	
	Male	1.37 (1.04–1.82)	0.03	1.47 (1.20–1.79)	< 0.001	1.93 (1.05–3.55)	0.03
Residence	Rural	Ref.		Ref.		Ref.	
	Urban	1.16 (0.83–1.61)	0.38	0.95 (0.72–1.25)	0.72	0.62 (0.30–1.31)	0.21
Maternal level of education	No education	Ref.		Ref.		Ref.	
	Primary	2.81 (1.27–6.21)	0.01	1.09 (0.75–1.60)	0.64	1.18 (0.24–5.77)	0.84
	Secondary	2.99 (1.34–6.65)	0.01	1.03 (0.71–1.50)	0.88	2.24 (0.50–10.16)	0.29
	Post‐secondary	2.40 (0.98–5.87)	0.06	1.08 (0.69–1.68)	0.75	0.79 (0.13–4.83)	0.80
Shared toilet	No	Ref.		Ref.		Ref.	
	Yes	1.04 (0.77–1.42)	0.79	1.27 (1.02–1.57)	0.04	0.90 (0.45–1.83)	0.78
Sex of the household head	Female	Ref.		Ref.		Ref.	
	Male	0.97 (0.63–1.51)	0.91	1.53 (0.97–2.42)	0.07	1.14 (0.44–2.97)	0.78
Maternal media exposure	Not exposed	Ref.		Ref.		Ref.	
	Exposed	0.80 (0.58–1.11)	0.19	1.11 (0.88–1.27)	0.79	1.30 (0.69–2.44)	0.42
Type of toilet	Unimproved	Ref.		Ref.		Ref.	
	Improved	0.97 (0.71–1.34)	0.87	1.03 (0.83–1.27)	0.79	0.89 (0.40–1.98)	0.78
Wealth index	Poorest	Ref.		Ref.		Ref.	
	Poor	0.97 (0.64–1.46)	0.87	1.04 (0.78–1.39)	0.79	1.95 (0.74–5.15)	0.18
	Middle	0.84 (0.52–1.36)	0.48	0.91 (0.66–1.26)	0.59	1.26 (0.42–3.79)	0.68
	Rich	0.86 (0.53–1.42)	0.57	0.89 (0.63–1.25)	0.49	1.86 (0.63–5.47)	0.28
	Richest	0.61 (0.33–1.12)	0.11	1.03 (0.69–1.54)	0.88	1.37 (0.39–4.83)	0.63
Stunting	Absent	Ref.		Ref.		Ref.	
	Present	1.13 (0.80–1.61)	0.48	1.22 (0.96–1.56)	0.11	0.76 (0.33–1.80)	0.54
Wasting	Absent	Ref.		Ref.		Ref.	
	Present	1.33 (0.81–2.18)	0.27	0.89 (0.62–1.28)	0.52	0.76 (0.26–2.22)	0.61
Underweight	Absent	Ref.		Ref.		Ref.	
	Present	0.88 (0.58–1.35)	0.56	1.20 (0.90–1.60)	0.21	1.48 (0.59–3.71)	0.41

**FIGURE 3:**
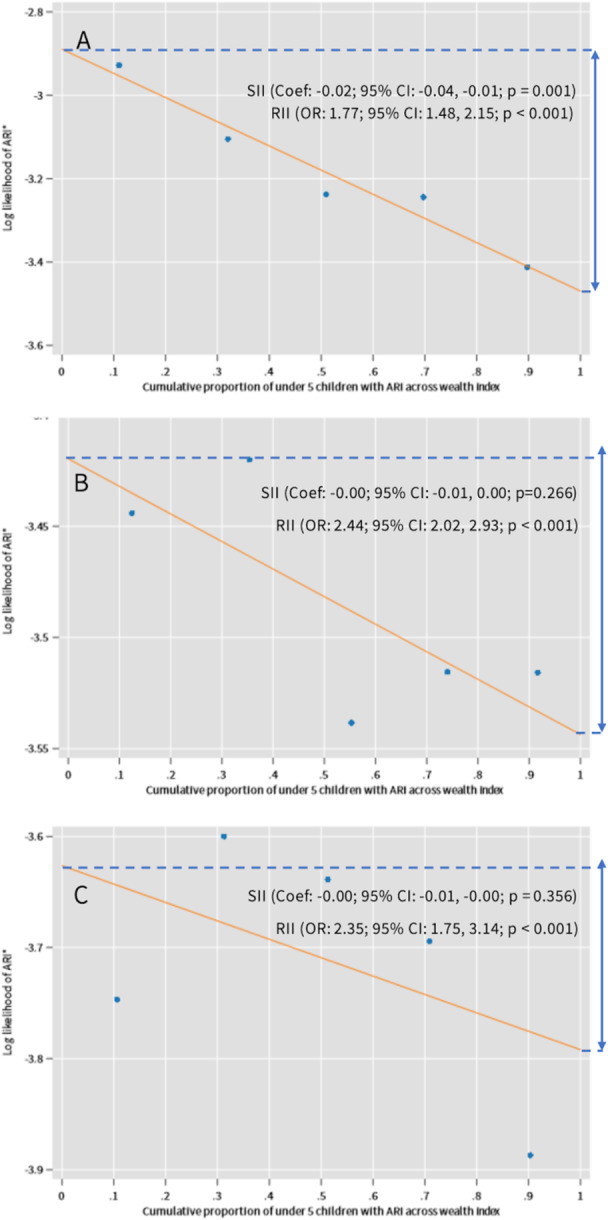
Slope index inequality (SII) and relative index inequality (RII) of ARI: (A) 2017–2018; (B) 2019; and (C) 2022.

Figure 3 shows wealth‐related inequality in ARI across survey years. In 2017–2018, both absolute and relative inequality were significant (SII: Coef.: −0.02, 95% CI: −0.04, −0.01, *p* = 0.001; RII: OR: 1.77, 95% CI: 1.48, 2.15, *p* < 0.001). In 2019, absolute inequality was not significant, while relative inequality remained significant (RII: OR: 2.44, 95% CI: 2.02, 2.93, *p* < 0.001). In 2022, absolute inequality was again not significant, but relative inequality was (RII: OR: 2.35, 95% CI: 1.75, 3.14, *p* < 0.001) (Figure 3).

Supporting Figure 1 shows that all significant predictors of ARI, including child's age, parental age, sex, wealth index, and stunting, had consistent odds ratios across survey years. No heterogeneity was detected, indicating that the strength and direction of associations remained stable over time (Supporting Figure 1).

Younger children consistently had higher prevalence of diarrhoea than older children in all years (*p* < 0.001). Parental age was associated in 2017–2018 and 2019 (*p* < 0.001 for both parents), but not in 2022. Male sex was significant only in 2017–2018 (57.1% vs. 51.8%, *p* = 0.02). Maternal education showed no difference in 2017–2018; but in 2019, children of mothers with primary education had a higher prevalence (26.7% vs. 12.9% among post‐secondary, *p* = 0.001); no association was observed in 2022. Household sanitation factors were significant in 2019, where shared toilet use (*p* = 0.001) and unimproved toilets (*p* = 0.004) were associated with higher prevalence, but these associations were absent in 2017–2018 and 2022. Wealth status showed no effect in 2017–2018 but was significant in 2019 (30.8% in poorest vs. 12.3% in richest, *p* < 0.001) and in 2022 (23.9% vs. 18.8%, *p* = 0.01). Maternal media exposure was linked with higher prevalence in 2017–2018 (68.3% vs. 63.8%, *p* = 0.06), but with lower prevalence in 2019 (55.9% vs. 63.0%, *p* < 0.001). Nutritional factors were significant only in 2019: underweight (27.4% vs. 22.4%, *p* < 0.001) and wasting (11.7% vs. 9.7%, *p* = 0.01), while stunting showed borderline association (*p* = 0.05) (Table 3).

**TABLE 3:** Frequency and distribution of factors associated with diarrhoea.

Variables	BDHS 2017–2018			MICS 2019			BDHS 2022		
	Total	Diarrhoea	*p* value	Total	Diarrhoea	*p* value	Total	Diarrhoea	*p* value
	*n* (%)	Yes, *n* (%)*		*n* (%)	Yes, *n* (%)*		*n* (%)	Yes, *n* (%)*	
Child's age in months	28.0 (14.0–44.0)	18.0 (11.0–29.0)	< 0.001	30.0 (15.0–44.0)	21.0 (11.0–35.0)	< 0.001	29.0 (13.0–44.0)	21.0 (10.3–35.0)	< 0.001
Mother's age in years	25.0 (21.0–30.0)	24.0 (21.0–28.0)	< 0.001	26.0 (23.0–31.0)	26.0 (22.0–30.0)	< 0.001	26.0 (22.0–30.0)	25.0 (22.0–30.0)	0.04
Father's age in years (*n* = 8172)	33.0 (29.0–38.0)	31.0 (28.0–36.0)	< 0.001	34.0 (30.0–39.0)	33.0 (29.0–38.0)	< 0.001	34.0 (29.0–39.0)	34.5 (29.0–39.0)	0.83
Household head's age in years	39.0 (30.0–54.0)	37.5 (30.0–54.8)	0.09	38.0 (32.0–52.0)	37.0 (30.0–48.0)	< 0.001	40.0 (32.0–54.0)	40.0 (32.0–55.0)	0.51
Number of U5C in the household	1.0 (1.0–2.0)	1.0 (1.0–2.0)	0.25	1.0 (1.0–2.0)	1.0 (1.0–2.0)	0.93	1.0 (1.0–2.0)	1.0 (1.0–2.0)	0.96
Sex (male), *n* (%)	4327 (52.1)	234 (57.1)	0.02	11,944 (51.7)	851 (53.6)	0.13	4129 (51.1)	218 (53.2)	0.42
Residence (urban), *n* (%)	2869 (34.5)	144 (35.1)	0.80	4301 (18.6)	298 (18.8)	0.89	2661 (33.0)	133 (32.4)	0.83
Maternal level of education			0.84			0.00			0.59
No education	591 (7.1)	33 (8.0)		2594 (11.2)	201 (12.7)		501 (6.2)	26 (6.3)	
Primary	2384 (28.7)	121 (29.5)		5559 (24.1)	424 (26.7)		1934 (24.0)	95 (23.2)	
Secondary	3915 (47.1)	189 (46.1)		11,351 (49.2)	758 (47.7)		4174 (51.7)	224 (54.6)	
Post‐secondary	1418 (17.1)	67 (16.3)		3581 (15.5)	205 (12.9)		1466 (18.2)	65 (15.9)	
Shared toilet (yes), *n* (=7279) (%)	2533 (34.8)	139 (37.4)	0.29	6388 (28.2)	494 (31.8)	0.00	2097 (29.0)	116 (31.4)	0.32
Sex of the household head (male), *n* (%)	7271 (87.5)	364 (88.8)	0.43	21,258 (92.1)	1483 (93.4)	0.05	7088 (87.8)	368 (89.8)	0.23
Maternal media exposure (exposed), *n* (%)	5318 (64.0)	280 (68.3)	0.06	14,303 (62.6)	881 (55.9)	< 0.001	4587 (56.8)	226 (55.1)	0.50
Type of toilet (improved), *n* (=7350) (%)	4725 (64.3)	242 (64.5)	0.92	15,224 (66.0)	994 (62.6)	0.004	5820 (80.1)	282 (76.2)	0.07
Wealth index			0.08			< 0.001			0.01
Poorest	1824 (22.0)	92 (22.4)		5753 (24.9)	489 (30.8)		1697 (21.0)	98 (23.9)	
Poor	1667 (20.1)	80 (19.5)		4833 (20.9)	379 (23.9)		1607 (19.9)	102 (24.9)	
Middle	1482 (17.8)	89 (21.7)		4352 (18.9)	257 (16.2)		1620 (20.1)	73 (17.8)	
Rich	1636 (19.7)	62 (15.1)		4306 (18.7)	267 (16.8)		1586 (19.6)	60 (14.6)	
Richest	1699 (20.5)	87 (21.2)		3841 (16.6)	196 (12.3)		1565 (19.4)	77 (18.8)	
Stunting (present), *n* (=7846) (%)	2461 (31.4)	106 (27.0)	0.06	5963 (27.2)	445 (29.3)	0.054	924 (23.7)	41 (19.6)	0.17
Wasting (present), *n* (=7828) (%)	667 (8.5)	32 (8.2)	0.80	2175 (9.9)	179 (11.7)	0.013	460 (11.8)	23 (11.1)	0.74
Underweight (present), *n* (=8047) (%)	1805 (22.4)	89 (22.3)	0.93	5113 (22.8)	426 (27.4)	< 0.001	904 (22.9)	41 (19.5)	0.25

* Column % may not add up to 100% due to rounding errors. Percentages in the outcome column represent the proportion of children with diarrhoea within each covariate category.

Across all surveys, older child age was consistently associated with lower odds of diarrhoea (2017–2018: aOR: 0.97, 95% CI: 0.97–0.98; 2019: aOR: 0.98, 95% CI: 0.97–0.98; 2022: aOR: 0.98, 95% CI 0.97–0.99). Father's age showed a modest positive association only in 2019 (aOR: 1.02, 95% CI: 1.00–1.03). Urban residence increased risk in 2019 (aOR: 1.26, 95% CI: 1.09–1.47) but not in other years. Wealth index was associated with lower odds in 2017–2018 for richer households (aOR: 0.62, 95% CI: 0.40–0.96) and lower odds in 2019 for middle (aOR: 0.73, 95% CI: 0.61–0.88), rich (aOR: 0.78, 95% CI: 0.65–0.95), and richest (aOR: 0.64, 95% CI: 0.51–0.81) quintiles. Maternal media exposure showed opposite effects: higher odds in 2017–2018 (aOR = 1.44, 95% CI 1.10–1.90) and lower odds in 2019 (aOR = 0.85, 95% CI 0.75–0.97), with no effect in 2022. Nutritional factors showed an association only in 2019, where underweight children had higher odds (aOR = 1.34, 95% CI 1.14–1.57) (Table 4).

**TABLE 4:** Logistic regression of factors associated with diarrhoea.

Variable	Categories	BDHS 2017–2018 (*n* = 6633)		MICS 2019		BDHS 2022	
		aOR (95% CI)	*p* value	aOR (95% CI)	*p* value	aOR (95% CI)	*p* value
Child's age in months		0.97 (0.97–0.98)	< 0.001	0.98 (0.97–0.98)	< 0.001	0.98 (0.97–0.99)	< 0.001
Mother's age in years		0.97 (0.95–0.98)	0.08	0.98 (0.96–0.99)	< 0.001	0.99 (0.95–1.03)	0.64
Father's age in years		1.00 (0.98–1.02)	0.99	1.02 (1.00–1.03)	0.01	1.02 (0.99–1.05)	0.14
Household head's age in years		0.99 (0.99–1.01)	0.83	0.99 (0.98–0.99)	0.00	1.01 (0.99–1.02)	0.31
Number of U5C in the household		0.96 (0.81–1.15)	0.68	0.9 (0.89–1.10)	0.81	0.84 (0.65–1.09)	0.18
Sex	Female	Ref.		Ref.		Ref.	
	Male	1.22 (0.98–1.52)	0.07	1.06 (0.95–1.18)	0.31	1.11 (0.82–1.49)	0.49
Residence	Rural	Ref.		Ref.		Ref.	
	Urban	0.99 (0.76–01.28)	0.92	1.26 (1.09–1.47)	0.003	1.11 (0.78–1.58)	0.58
Maternal level of education	No education	Ref.		Ref.		Ref.	
	Primary	0.78 (0.50–1.21)	0.26	1.00 (0.82–1.22)	0.99	1.18 (0.59–2.35)	0.64
	Secondary	0.76 (0.49–1.18)	0.21	0.95 (0.78–1.15)	0.58	1.04 (0.52–2.05)	0.92
	Post‐secondary	0.74 (0.44–1.24)	0.25	0.90 (0.70–1.15)	0.40	0.64 (0.28–1.43)	0.27
Shared toilet	No	Ref.		Ref.		Ref.	
	Yes	1.08 (0.85–1.38)	0.55	1.07 (0.94–1.21)	0.30	1.18 (0.84–1.66)	0.35
Sex of the household head	Female	Ref.		Ref.		Ref.	
	Male	1.02 (0.72–1.45)	0.89	1.03 (0.83–1.29)	0.79	0.99 (0.62–1.60)	0.97
Maternal media exposure	Not exposed	Ref.		Ref.		Ref.	
	Exposed	1.44 (1.10–1.90)	0.01	0.85 (0.75–0.97)	0.01	1.10 (0.79–1.52)	0.57
Type of toilet	Unimproved	Ref.		Ref.		Ref.	
	Improved	1.04 (0.80–1.34)	0.78	0.92 (0.82–1.03)	0.129	1.22 (0.80–1.85)	0.37
Wealth index	Poorest	Ref.		Ref.		Ref.	
	Poor	0.88 (0.64–1.32)	0.50	0.98 (0.84–1.15)	0.87	1.27 (0.80–2.02)	0.30
	Middle	1.12 (0.78–1.63)	0.56	0.73 (0.61–0.88)	< 0.001	0.76 (0.45–1.29)	0.31
	Rich	0.62 (0.40–0.96)	0.03	0.78 (0.65–0.95)	0.011	0.85 (0.50–1.48)	0.58
	Richest	1.00 (0.63–1.59)	0.99	0.64 (0.51–0.81)	< 0.001	0.95 (0.52–1.77)	0.88
Stunting	Absent	Ref.		Ref.		Ref.	
	Present	0.84 (0.62–1.12)	0.24	0.99 (0.86–1.14)	0.87	0.89 (0.58–1.39)	0.62
Wasting	Absent	Ref.		Ref.		Ref.	
	Present	0.85 (0.55–1.30)	0.45	0.97 (0.80–1.18)	0.78	0.94 (0.54–1.64)	0.82
Underweight	Absent	Ref.		Ref.		Ref.	
	Present	1.28 (0.91–1.80)	0.15	1.34 (1.14–1.57)	< 0.001	0.97 (0.59–1.59)	0.89

**FIGURE 4:**
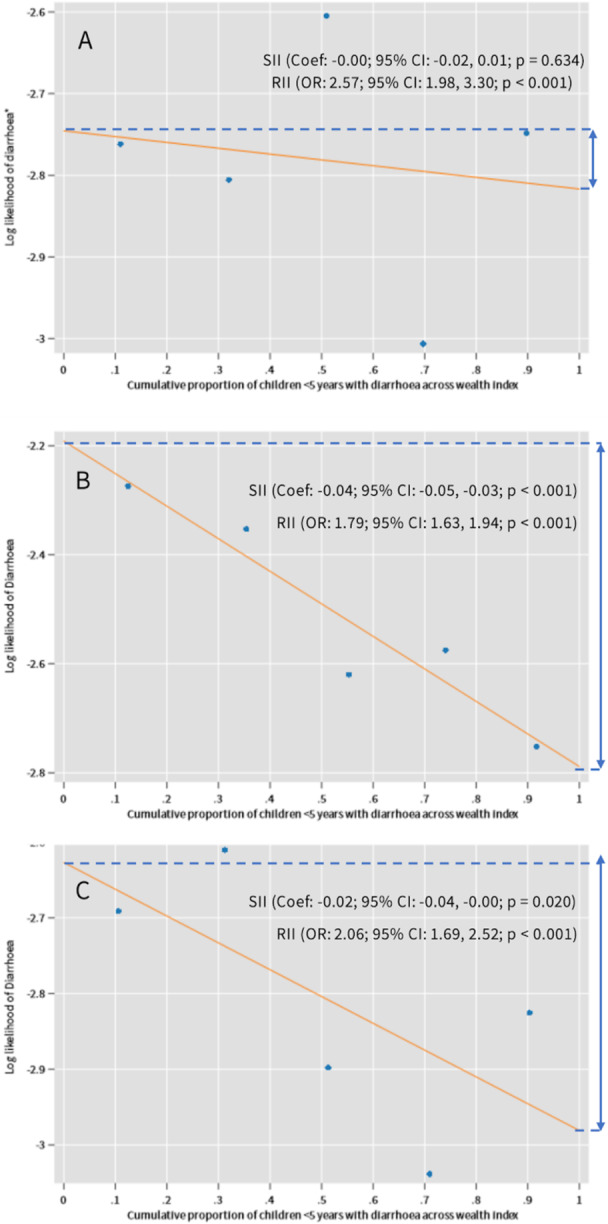
Slope index inequality (SII) and relative index inequality (RII) of diarrhoea: (A) 2017–2018; (B) 2019; and (C) 2022.

Figure 4 shows wealth‐related inequality in diarrhoea across survey years. In 2017–2018, absolute inequality was not significant, but relative inequality was strong (RII: OR: 2.57, 95% CI: 1.98, 3.30, *p* < 0.001). In 2019, both absolute and relative inequality were significant (SII: Coef.: −0.04, 95% CI: −0.05, −0.03, *p* < 0.001; RII: OR: 1.79, 95% CI: 1.63, 1.94, *p* < 0.001). In 2022, both were significant (SII: Coef: −0.02, 95% CI: −0.04, −0.00, *p* = 0.020; RII: OR: 2.06, 95% CI: 1.69, 2.52, *p* < 0.001).

Supporting Figure 2 shows that all significant predictors of diarrhoea, including child's age, mother's age, sex, maternal education, household head's sex, media exposure, toilet type, and wealth index, had consistent odds ratios across survey years. Only the underweight showed heterogeneity, indicating variation in effect between surveys (Supporting Figure 2).

## 4 Discussion

This study assessed the trends, prevalence, and factors associated with the two leading causes of under‐5 mortality in Bangladesh, that is, ARI and diarrhoea, using the BDHS 2017–2018, MICS 2019, and BDHS 2022 surveys [9]. Findings showed a significant downward linear trend in ARI prevalence. Previous literature supports that ARI has declined over the past two decades due to a combination of efforts, including expanded programmes on immunisations (EPI) and improved care‐seeking behaviours [24].

The diarrhoea prevalence fluctuated between 2017–2018 and 2022, peaking at around 6.9% in 2019. Previous studies have found similar findings, attributing the peak in 2019 to population growth, as it may have negative effects on clean water, sanitation, and poor health infrastructures [8]. The timing of the surveys indicates that the COVID‐19 pandemic may not be a major contributor, although suggested by other studies [8]. Moreover, the higher prevalence of diarrhoea observed in 2019 may partly reflect the timing of survey data collection. MICS 2019 was conducted between January and June, when diarrhoeal disease typically reaches its seasonal peak. In contrast, BDHS 2017–2018 was conducted from October to March, when diarrhoea prevalence is generally lowest. BDHS 2022 was conducted from June to December, which may have averaged out seasonal variation. These differences in survey timing likely contributed to the higher diarrhoea prevalence captured in 2019 compared with other survey years.

Additionally, consistent with earlier literature, male sex was associated with higher odds of ARI, while younger age was significantly associated with diarrhoea [3]. This higher odds for ARI in male children may be due to a stronger immune response in females [25]. This suggests that more interventions need to target younger male children who could be at greater risk for both diseases. Higher maternal education was shown to be associated with higher odds of ARI only in BDHS 2017–2018. Earlier studies reported that mothers who were more educated demonstrated a clearer perception and greater understanding of the nutritional and medical needs of their child thus offering one explanation as to why higher maternal education was significantly associated with higher odds of ARI [26]. The MICS 2019 and BDHS 2022 on the other hand did not show significantly higher odds of ARI with higher maternal education which can be explained by a national decrease in the proportion of uneducated mothers and an increase in mothers attaining higher education. This contributes to a lower protective effect on maternal education [27]. Another study in India that tracked under‐5 mortality rates explained a similar variation in the protective effect of maternal education as a result of other factors such as convergence in healthcare access across rural and urban areas [28].

In our study, underweight was associated with higher odds of diarrhoea in 2019 only. A previous study found a similar upsurge in diarrhoea among underweight children, with prevalence rising from 3.9% in 2012 to 6.9% in 2019 [14]. The seasonal variation caused by the data collection period may account for this finding.

On the other hand, the wealth index across all three survey years was associated with lower odds of both ARI and diarrhoea, particularly in the middle to richest quintiles. This finding supports previous literature that also found a higher risk of ARI and diarrhoea among children from middle, poor, and poorest compared to the richest [29]. The wealth index was more strongly associated with lower odds of diarrhoea in 2019 compared with other survey years. This may be related to survey timing, as MICS 2019 overlapped with the period when exposure risks are highest. During periods of heightened transmission, wealth‐related advantages such as improved housing, sanitation, and water access may provide greater protection, widening the gap between poorer and richer households [15].

The SII and RII analysis revealed that RII remained statistically significant for both ARI and diarrhoea throughout 2017–2018, 2019, and 2022, even as overall ARI prevalence declined. This suggests that improvements in disease burden might not have been equally distributed, and structural gaps between wealth groups exist. This is consistent with existing evidence that found childhood ARI and diarrhoeal morbidity to be disproportionately concentrated among socioeconomically disadvantaged households using BDHS data from 1993 to 2014 [30]. Therefore, from 2017 to 2022, these structural gaps between wealth groups still continue to persist. Absolute inequality, on the other hand, was significant only in certain years (2017–2018 for ARI and 2022 for diarrhoea), indicating shifts in the magnitude but not the direction of disparity. Beyond persistent inequality, the distribution of ARI and diarrhoea across wealth groups suggests possible shifts in burden over time. While ARI declined overall, sustained relative inequality indicates uneven gains across socioeconomic groups. For diarrhoea, variation across survey years may reflect changing burden between poorer and better‐off households. Targeted interventions for disadvantaged groups, including improvements in sanitation and access to clean household energy, may reduce disease burden [31].

Temporal changes in ARI and diarrhoea prevalence should be interpreted considering Bangladesh's existing health and environmental policies. Diarrhoea control has been supported through nationwide promotion of oral rehydration salts, zinc supplementation, and WASH‐related improvements [32]. ARI management has largely been addressed through the Integrated Management of Childhood Illness strategy, which demonstrated substantial improvements in child illness case management, care‐seeking, and feeding practices [33]. In addition, broader environmental and social improvements, including sanitation, hygiene, and nutritional progress, likely intersect with these policies in reducing childhood diarrhoeal disease burden.

Strengths of this study include the use of three nationally representative datasets with large sample sizes and standardised survey tools, allowing robust comparisons across years. The analysis examined both ARI and diarrhoea simultaneously, providing insights into shared and distinct determinants. Additional strengths are the use of pooled analyses, inequality measures (SII and RII), and homogeneity tests, which strengthen the reliability and policy relevance of the findings.

However, limitations include the cross‐sectional design, which precludes causal inference, and reliance on self‐reported symptoms, which may introduce recall bias and outcome misclassification. Missing data and complete case analysis may have introduced selection bias. Differences between BDHS and MICS in measurement and implementation may affect comparability, and residual confounding from unmeasured factors such as environmental exposures and healthcare access cannot be excluded. Data were limited to 2022. Differences in survey timing may have influenced diarrhoea prevalence estimates, as MICS 2019 was conducted during the pre‐monsoon and early monsoon months, when diarrhoea typically peaks, whereas BDHS 2017–2018 and BDHS 2022 were conducted during periods of lower transmission. Seasonal variation in data collection likely contributed to the higher prevalence observed in 2019. Sampling weights were not applied due to pooling of multiple surveys with differing designs, which may affect the national representativeness of the estimates.

## 5 Conclusions

Across three nationally representative surveys, ARI prevalence declined steadily, while diarrhoea prevalence fluctuated, peaking in 2019. Male sex was consistently associated with higher odds of ARI, and younger child age was associated with diarrhoea. The wealth index showed lower odds for both conditions, though relative inequality persisted across all survey years. Underweight was associated with diarrhoea only in 2019, likely reflecting seasonal variation in survey timing. Despite overall progress, inequities by sex, age, nutrition, and household wealth remain evident. Future interventions should prioritise younger male children and households with fewer resources, while ensuring equitable access to prevention and care. Continued monitoring is required for achieving further reductions in under‐5 morbidity and mortality in Bangladesh.

## Author Contributions

**Mahdia Ahmed:** conceptualisation, methodology, writing – original draft, data curation, formal analysis. **Md Fuad Al Fidah:** methodology, formal analysis, writing – original draft, data curation. **Mahabub Uz Zaman:** methodology, writing – original draft. **Md Ridwan Islam:** writing – original draft, formal analysis, methodology. **Tahmeed Ahmed:** conceptualisation, writing – review and editing. **Mustafa Mahfuz:** supervision, writing – review and editing, conceptualisation.

## Funding

The authors have nothing to report.

## Ethics Statement

The current study used data from a publicly available website. Since secondary analysis was conducted, no ethical clearance was required. All original data collection received ethical clearance (including written informed consent obtained from participants or their legal guardians during the original surveys) from National Review Boards.

## Conflicts of Interest

The authors declare no conflicts of interest.

## Transparency Statement

The lead author Md Fuad Al Fidah affirms that this manuscript is an honest, accurate, and transparent account of the study being reported; that no important aspects of the study have been omitted; and that any discrepancies from the study as planned (and, if relevant, registered) have been explained.

## Supporting information

Supporting File

## Data Availability

Data related to this manuscript are publicly available at, https://mics.unicef.org/surveys and https://dhsprogram.com/data/available-datasets.cfm on registration.
