# Supplementary material for: Exploration of the Temporal Trends, Prevalence, and Factors of Acute Respiratory Infection or Diarrhoea: A Cross‐Sectional Analysis of Three National Surveys
Source: Health Sci Rep. 2026 May 13;9(5):e72529. doi: 10.1002/hsr2.72529 (PMC13169161; doi:10.1002/hsr2.72529)
Supplement: Supplementary file 1 — Supporting File [file HSR2-9-e72529-s001.docx]

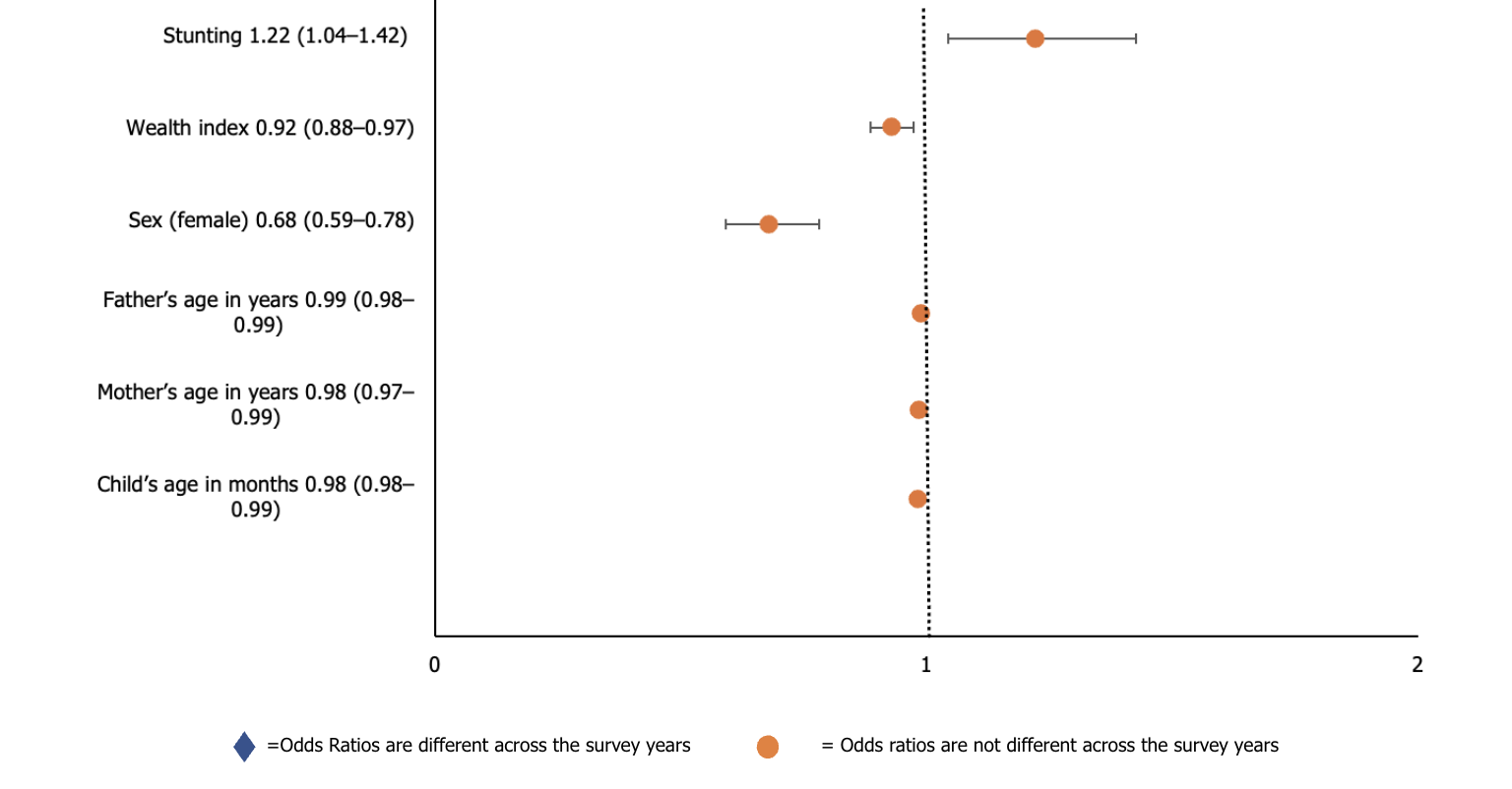


**Supplementary Figure 1. Pooled odds ratios from adjusted for survey years (Mantel-Hanszel test) and approximate test of homogeneity of odds ratios for ARI**


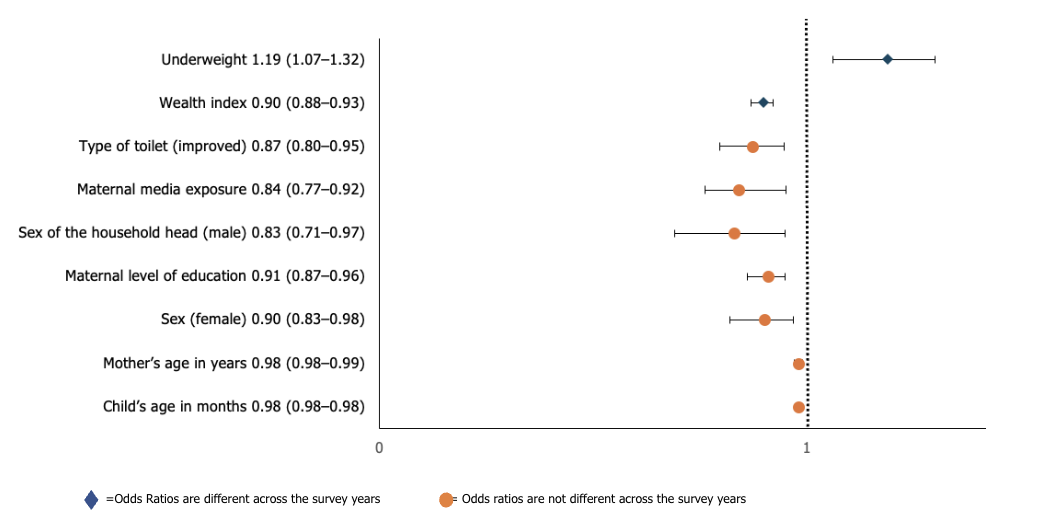


**Supplementary Figure 2. Pooled odds ratios adjusted for survey years (Mantel-Haenszel test) and approximate test of homogeneity of odds ratios of all significant variables associated with diarrhoea**
